# Heterofucan from *Sargassum filipendula* Induces Apoptosis in HeLa Cells

**DOI:** 10.3390/md9040603

**Published:** 2011-04-14

**Authors:** Leandro Silva Costa, Cinthia Beatrice Silva Telles, Ruth Medeiros Oliveira, Leonardo Thiago Duarte Barreto Nobre, Nednaldo Dantas-Santos, Rafael Barros Gomes Camara, Mariana Santana Santos Pereira Costa, Jailma Almeida-Lima, Raniere Fagundes Melo-Silveira, Ivan Rui Lopes Albuquerque, Edda Lisboa Leite, Hugo Alexandre Oliveira Rocha

**Affiliations:** 1 Laboratory of Biotechnology of Natural Polymers (BIOPOL), Departament of Biochemistry, Federal University of Rio Grande do Norte (UFRN), Natal-RN, Brazil; E-Mails: cinthiatelles@yahoo.com.br (C.B.S.T), rmo_85@hotmail.com (R.M.O.); leo_dnobre@yahoo.com.br (L.T.D.B.N.); nednaldod@hotmail.com (N.D.-S.); rafael_bgc@yahoo.com.br (R.B.G.C.); marispc_bio@yahoo.com.br (M.S.S.P.C.); biolottus23@yahoo.com.br (J.A.-L.); ranierefagundes@hotmail.com (R. F.M.-S.); ivan.rui@click21.com.br (I.R.L.A.); eddaleite@cb.ufrn.br (E.L.L.); 2 Federal Institute of Education, Science and Technology of Rio Grande do Norte (IFRN), Santa Cruz-RN, Brazil; E-Mail: leandro-silva-costa@hotmail.com (L.S.C.)

**Keywords:** fucoidan, sulfated polysaccharides, anticancer, apoptosis-inducing factor (AIF)

## Abstract

Fucan is a term used to denominate a family of sulfated polysaccharides rich in sulfated l-fucose. Heterofucan SF-1.5v was extracted from the brown seaweed *Sargassum filipendula* by proteolytic digestion followed by sequential acetone precipitation. This fucan showed antiproliferative activity on Hela cells and induced apoptosis. However, SF-1.5v was not able to activate caspases. Moreover, SF-1.5v induced glycogen synthase kinase (GSK) activation, but this protein is not involved in the heterofucan SF-1.5v induced apoptosis mechanism. In addition, ERK, p38, p53, pAKT and NFκB were not affected by the presence of SF-1.5v. We determined that SF-1.5v induces apoptosis in HeLa mainly by mitochondrial release of apoptosis-inducing factor (AIF) into cytosol. In addition, SF-1.5v decreases the expression of anti-apoptotic protein Bcl-2 and increased expression of apoptogenic protein Bax. These results are significant in that they provide a mechanistic framework for further exploring the use of SF-1.5v as a novel chemotherapeutics against human cervical cancer.

## 1. Introduction

Carcinoma of the uterine cervix is the second most common female tumor worldwide, surpassed only by breast cancer and its incidence is disproportionately high (>80%) in the developing world. Primary treatment can be either surgery or a combination of radiotherapy/chemotherapy for early-stage patients. Treatment of distant disease is usually palliative, aimed at symptom control. Targeted radiotherapy may be useful for controlling local symptoms. While chemotherapy may sometimes shrink tumor masses, there is no survival advantage [1]. Better and more effective chemotherapeutics are apparently needed for these patients to improve survival rates.

Evidence has accumulated in recent years, showing that many cancer chemotherapeutic agents kill cancer cells by inducing cell death. Cells die in a process that is reversible until a first irreversible phase or ‘point-of-no-return’ is reached, but this is not a clearly defined biochemical event [2]. Thus, identifying the mode of cell death is recognized as a novel strategy for screening anticancer drugs. As a very valuable source for novel chemotherapeutic reagents, active sulfated homo-heterofucans isolated from the brown seaweed have shown effective antitumor activities with a wide range of mechanisms [3].

Fucan is a term used to define a family of l-fucose-containing sulfated polysaccharides found in brown seaweed and several species of echinoderms, mostly from the egg jelly of sea urchins [4]. The structures of these fucans vary among species and sometimes among different parts of the seaweed [5]. Furthermore, in contrast to animal fucans, algal fucans may have portions of other neutral sugars and uronic acids in addition to sulfate and fucose in their structures. Some algal fucans exhibit important pharmacological activities such as anticoagulant [6], antipeptic [7], anticomplementary, antiinflammatory, antiviral [3], antiadhesive [8], antiproliferative [9], antioxidant [10] and apoptosis-inducing [11,12]. As a result, fucans have a multitude of potential applications in human health care. Additionally, biomaterials derived from seaweed generally have an advantage in that there is no potential risk of contamination from animal viruses and bovine spongiform encephalopathy (BSE) pathogens [13].

In a program aimed at determining the bioactivity of sulfated polysaccharides from tropical brown seaweeds, we found that the polysaccharide-rich extract from *Sargassum filipendula* C.Agardh showed significant antiproliferative effect on HeLa cell (human uterine adenocarcinoma cell line) proliferation [10]. In the preceding article a bioassay-guided fractionation of this extract led to the isolation of an antioxidant heterofucan denominated SF-1.5v, which exhibits antiproliferative activity against HeLa cells. However, the molecular mechanism underlying the SF-1.5v-induced antiproliferative process remains unclear.

The primary objective of this study was to determine the relevant mechanisms for an antiproliferative effect of the heterofucan SF1.5v. We determined that SF-1.5v induces apoptosis in HeLa mainly by releasing the apoptosis-inducing factor (AIF) from mitochondria into cytosol. These results are significant in that they provide a mechanistic framework for further exploring the use of SF-1.5v as a novel chemotherapeutics for human cervical cancer.

## 2. Results and Discussion

### 2.1. Growth Inhibition by Heterofucan SF-1.5v

**Figure 1.**
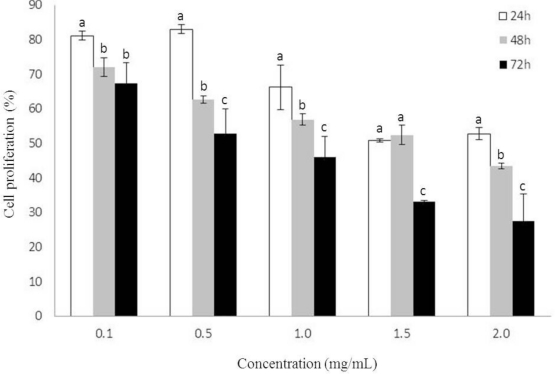
HeLa cell proliferation in the presence of sulfated polysaccharide from *Sargassum filipendula.* Each value is the mean ±SD of seven determinations. ^a,b,c^ Indicate a significant difference (p < 0.05) between treatments at the same concentration.

We studied the inhibitory effect of heterofucan SF-1.5v (from 0.1 to 2.0 mg/mL) on the proliferation of HeLa cells cultured for 24, 48 and 72 hours. Figure 1 displays MTT assay results as a measure of cell growth. Proliferation is presented as a percentage of cell proliferation under no treatment conditions. A significant time and dose dependent decrease in cell proliferation was observed. The effect was significant at 24 hours, but optimized at 72 hours (Figure 1), showing antiproliferative activity between 32.7% and 72.5% at concentrations from 0.1 to 2.0 mg/mL.

Antiproliferative activity of the heterofucan SF-1.5v was considerably higher than that of fucans from *Sargassum kjellmanianum* and *Sargassum stenophyllum*, which showed no more than 40% inhibition activity on the growth of L-1210 leukemia and HeLa cells, respectively [14,15]. These considerable variations in antiproliferative activity between fucans likely result from the various chemical compositions of fucan polymers originating in the different species, anatomical regions, growing conditions of brown seaweeds, extraction and purification procedures as well as the use of different cancer cell lines.

### 2.2. Heterofucan SF-1.5v-Induced Apoptosis in HeLa Cells

**Figure 2.**
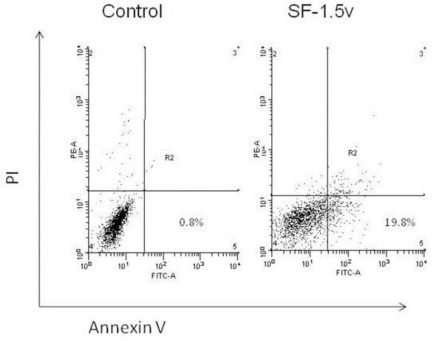
Flow cytometry analysis of apoptotic death of HeLa cells by SF-1.5v. Dot plots display the apoptotic death of HeLa cells treated with 1.5 mg/ml of SF-1.5v. Annexin−/PI− (LL), viable cells; Annexin+/PI− (LR), cells undergoing apoptosis; Annexin+/PI+ (UR), cells that are in end-stage apoptosis or are already dead. LL, lower left; LR, lower right; UR, Upper right. One representative FACS assay of three independent experiments is presented. The percentage in LR corresponds to Annexin+/PI− cells.

A typical assay was performed to characterize whether cell death resulting from fucan treatment was caused by apoptosis induction. Thus, we examined the effect of the fucan (1.5 mg/mL) on apoptosis using annexin V/PI double staining. One of the early features of cells undergoing apoptosis is phosphatidylserine (PS) translocation from the inner to the outer leaflet of the plasma membrane. Figure 2 shows PI *vs.* annexin V-FITC fluorescence. The lower right quadrants represent the early apoptotic cells: annexin V binding and PI negative. Annexin V and PI staining revealed that SF-1.5v increased apoptosis compared to the control.

### 2.3. Heterofucan SF-1.5v Treatment-Induced Apoptosis Did Not Require Activation of Caspases in HeLa Cells

**Figure 3.**
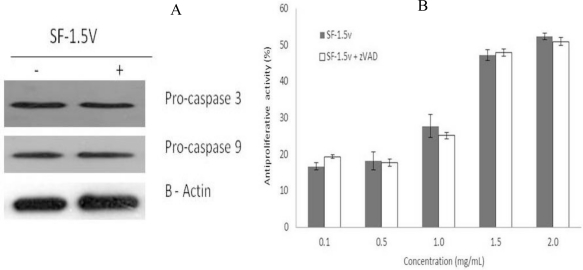
Heterofucan SF-1.5v treatment-induced apoptosis did not require caspase activation in HeLa cells. (**A**) Effects of SF-1.5v in activation of upstream caspase-9 and of downstream caspase-3. One representative immunoblot of three independent experiments is presented. (**B**) Caspase inhibitor z-VAD (50mM) does not suppress SF-1.5v-induced apoptosis in HeLa cells. Each value is the mean ± SD of seven determinations.

As the family of aspartate-specific cysteinyl proteases (caspases) plays a pivotal role in the execution of programmed cell death, we determined whether the apostosis induction by the heterofucan SF-1.5v resulted in activation of caspase-9 and caspase-3. Caspase activations were measured using western blot analysis. Cells received no treatment (control) or were treated with heterofucan SF-1.5v (1.5 mg/mL) for 24 hours. In response to the heterofucan, the activation of pro-caspase-9 and pro-caspase-3 did not increase (Figure 3A). In order to rule out caspase participation in SF-1.5v-induced apoptosis, the cells were incubated with SF-1.5v (from 0.1 to 2.0 mg/mL) in the presence of pan-caspase inhibitor z-VAD (50 mM) for 24 hours. In every condition this compound failed to inhibit SF-1.5v-induced apoptosis (Figure 3B), indicating that caspase activation is not essential for heterofucan SF-1.5v-induced apoptosis in HeLa cells.

Although several studies show fucans inducing cell death by caspase activation, it is known that this does not necessarily lead to apoptosis and that caspase inhibition does not necessarily prevent cell death, suggesting that caspase blockade or inactivation is not always a useful therapeutic target [2]. Additionally, some articles show fucans inducing death by mechanisms independent of caspases. For example, Aisa and colleagues [12] showed a fucan from *Fucus vesiculosus* inducing human lymphoma HS-Sultan cell death through activation of ERK pathways. Hyun and colleagues provide evidence demonstrating that the pro-apoptotic effect of this fucan from *F. vesiculosus* is mediated through ERK and p38 activation, and blocking of the PI3K/Akt signal pathway in HCT-15 colon carcinoma cells [16]. Moreover, *F. vesiculosus* fucan also affects the NFκB pathway [17]. Overall, fucans inhibit cell proliferation by affecting different survival pathways depending on cell type. Thus, HeLa cells were treated with heterofucan SF-1.5v (1.5 mg/mL) for 24 hours, after we investigated whether heterofucan SF-1.5v affects NFκB, Akt-GSK-3β and MAPK (ERK and p38) pathways by western blot.

**Figure 4.**
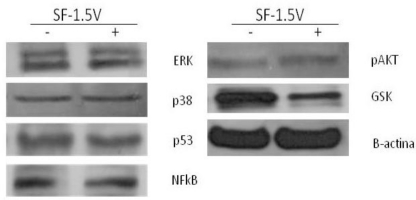
Analyses of the intracellular signaling of SF-1.5v by western blot. HeLa cells were treated with 1.5 mg/mL SF-1.5v for 24 hr. Phosphorylation of ERK, p38, p53, NFκB, Akt, and GSK was analyzed using phosphospecific antibodies. Each membrane was re-probed with anti-ERK, p38, p53, NFκB, Akt, and GSK antibodies to confirm equal protein loading. One representative immunoblot of three independent experiments is presented.

The heterofucan SF-1.5v did not affect phophorylation of ERK, p38 and NFκB proteins. On the other hand, SF-1.5v induces dephosphorylation (activation) of glycogen synthase kinase-beta (GSK-3β) (Figure 4). Since GSK is involved in activation of p53 [18], we also evaluated the phosphorylation of this protein. However, it was not affected by SF-1.5v (Figure 4). GSK Dephosphorylation is involved in apoptosis [19], indicating that SF-1.5v likely induces cell death, mainly by GSK activation.

### 2.4. Heterofucan SF-1.5v Treatment-Induced Apoptosis in the Presence of GSK Inhibitor

**Figure 5.**
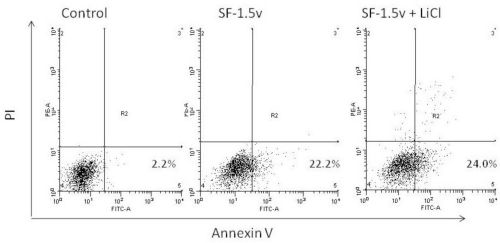
GSK inhibition by lithium chloride. HeLa cells were pretreated with or without 10 mM of GSK-specific inhibitor lithium chloride (LiCl), for 1 hr followed by incubation with 1.5 mg/mL of fucoidan for 24 hr. Apoptosis detection by annexin-V/PI staining was performed, as shown in Figure 1. Similar results were obtained in three independent experiments. The percentage presented corresponds to Annexin+/PI− cells.

In order to determine the role of GSK in SF-1.5v-induced apoptosis, HeLa cells were incubated with lithium chloride (10mM), a GSK-specific inhibitor. This was followed by stimulation with 1.5 mg/mL SF-1.5v for 24 hours and FACS analysis (Figure 5). Lithium chloride failed to inhibit heterofucan SF-1.5v-induced apoptosis, indicating that GSK activation is not essential for apoptosis by SF-1.5v in HeLa cells. This result is consistent with that shown by Aisa and colleages. These authors showed that the fucan from *F. vesiculosus* promotes GSK dephosphorylation, but that this effect is not involved in fucan-induced death of human HS-sultan cells [12].

### 2.5. Heterofucan SF-1.5v Induces High Levels of Apoptosis-Inducing Factor (AIF) in Cytoplasm

**Figure 6.**
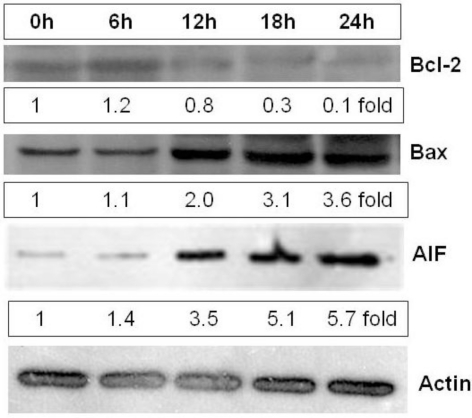
Analysis on AIF, Bax and Bcl-2 expression in the presence of SF-1.5v. HeLa cells were treated with 1.5 mg/mL SF-1.5v for 0, 6, 12, 18 and 24 hr. Levels AIF, Bax and Bcl-2 released into the cytosol was analyzed by immunoblotting using anti-AIF, anti-Bax or anti-Bcl-2 antibodies, as described in Materials and methods. In the boxes below the pictures there is the actin-adjusted level of AIF, Bax and Bcl-2. One representative immunoblot of three independent experiments is presented.

Several studies have shown that cell death is a caspase-independent self-destruction process activated by the mitochondrial pathway, an alternative programmed cell death pathway that occurs in the absence of caspase activation [2]. Permeabilization of the organelle that leads to the release of several proteins from the intermembrane space involved in organized cell death takes place in the mitochondrial pathway. The apoptosis-inducing factor (AIF), one of the soluble factors released from mitochondria, is able to force isolated nuclei to adopt apoptotic morphology in a caspase independent manner [20]. AIF is an FAD-containing, NADH-dependent oxidoreductase found in the mitochondrial intermembrane space. It induces phosphatidylserine exposure on the cell surface. It might also maintain apoptogenic ability in the presence of the pan-caspase inhibitor [21]. Given that heterofucan SF-1.5v induced phosphatidylserine exposure on the cell surface and maintained apoptogenic ability in the presence of the pan-caspase inhibitor, we analyzed the effect of this fucan on the amount of AIF in cytosol using western blotting. Figure 6 shows that AIF levels in cytosol are increased when cells were exposed to heterofucan SF-1.5v, indicating that the main SF-1.5v cell death mechanism is the mitochondrial release of AIF into the cytoplasm (Figure 6).

AIF is synthesized as a ∼67-kDa preprotein with an N-terminal extension and imported into mitochondria, where it is processed to the ∼62-kDa mature form. Topology analysis revealed that mature AIF is a type-I inner membrane protein with the N-terminus exposed to the matrix and the C-terminal portion to the intermembrane space. Upon induction of apoptosis, processing of mature AIF to a ∼57-kDa form occurred caspase-independently in the intermembrane space, releasing the processed form into the cytoplasm. Bcl-2 inhibited both these events [22]. In addition, the Bcl-2 tends to stabilize the barrier function of mitochondrial membranes, whereas proapoptotic Bax tend to antagonize such function and permeabilize the membranes. As Bcl-2 plays an integral role in the release of AIF during cell death, we determined its expression and correspondingly, also Bax in control and SF-1.5v-treated whole cell extracts. Western blot analysis clearly showed a suppression of Bcl-2 expression, accompanied by concomitant increases in Bax, in SF-1.5v-treated cells, compared to control cells (Figure 6). These results further support the ability of SF-1.5v to activate the mitochondria-dependent apoptotic cascade.

It is clear that fucans have a cell-dependent antitumor effect, since several articles have shown that fucans can inhibit the growth of some tumor cell lines, while not influencing the growth of others [3]. Moreover, the same fucan induces cell death in different cell lines by activating different cell-death signaling pathways, such as the fucan from *F. vesiculosus* [12,16]. However, this is the first report showing a fucan whose main antiproliferative mechanism is promoting the release of AIF from the mitochondria into the cytosol.

In the present study we showed that heterofucan SF-1.5v induces HeLa cell apoptosis by activating a cell-death mitochondrial pathway. It would therefore be interesting to investigate whether SF-1.5v can overcome the resistance of drug-refractory tumor cells. Additionally, the capacity of SF-1.5v to promote AIF release deserves further investigation, which might confirm SF-1.5v as a potential candidate for developing anticancer drugs for the treatment of human cervical cancer.

## 3. Experimental Section

### 3.1. Materials

Isopropanol, HCl and Tween 20 were obtained from Merck (Darmstadt, Germany). Deoxycholate, NaCl, EDTA, Na_3_VO_4_, NaF and protease inhibitors were purchased from Sigma-Aldrich Co. (St. Louis, USA). Cell culture medium components (Dulbecco's Modified Eagle Medium-DMEM), trypsin and fetal calf serum (FCS) were obtained from Cultilab (Campinas, Brazil). l-glutamine, sodium bicarbonate, sodium pyruvate and phosphate buffered saline (PBS) were purchased from Invitrogen Corporation (Burlington, ON, USA). All antibodies were purchased from Cell Signaling Technology (Danvers, MA, USA). The heterofucan SF-1.5v was obtained as described in the preceding article. All other solvents and chemicals were of analytical grade.

### 3.2. HeLa Cell Culture

HeLa cells were obtained from American Type Culture Collection (Manassas, VA, USA). HeLa cells were grown as previously described by Almeida-Lima *et al.* [23]. Briefly, HeLa cells were grown in DMEM medium supplemented with 10% fetal calf serum (FCS) (1% penicillin/streptomycin (10000 U/mL penicillin G sodium, 10000 mg/mL streptomycin sulfate). Cells were grown at 37 °C in a humidified 5% CO_2_ incubator. HeLa cells were seeded at a density of 5 × 10^6^ for 75 cm^3^ flasks.

### 3.3. Antiproliferative Activity

Antiproliferative activity of SF-1.5v was determined as previously described by Amoli *et al.* [24]. Briefly, HeLa cells were grown in 75 cm^3^ flasks in DMEM medium plus 10% FCS. Cells were seeded into 96-well plates at a density of 5 × 10^3^ cells/well and allowed to attach overnight in 300 μL medium FCS free incubated at 37 ºC, 5% CO_2_. The medium was then removed and 300 μL of medium/plus FCS was added, followed by heterofucan SF-1.5v at a final concentration of 0.1; 0.5; 1.0; 1.5 and 2.0 mg/mL. Cells growing under these conditions for 24 h, 48 h and 72 h at 37 ºC at 5% CO_2_. After incubation, traces of SF-1.5v were removed by washing the cells twice with 200 μL PBS and applying 100 μL of fresh medium and 10 μL of 12 mM MTT dissolved in PBS to determine the effects of the heterofucan on cell proliferation. Cells were then incubated for 4 h at 37 ºC, 5% CO_2_. To solubilize the product of MTT cleavage, 100 μL of isopropanol containing 0.04 N HCl was added to each well and thoroughly mixed using a multichannel pipettor. Within 1 h of HCl-isopropanol addition, absorbance at 570 nm was read using a Multiskan Ascent Microplate Reader (Thermo Labsystems, Franklin, MA, USA). The percent inhibition of cell proliferation was calculated as follows:

$$\frac{\%\;\text{Inhibition}=\text{Abs.}\;570\;\text{nm}\;\text{Control}-\text{Abs.}\;570\;\text{nm}\;\text{sample}}{\text{Abs.}\;570\;\text{nm}\;\text{Control}}\times 100$$

### 3.4. Apoptosis Assay

The apoptotic status of HeLa cells was evaluated by measuring the exposure of phosphatidylserine on cell membranes using annexin V-fluorescein isothiocyanate (annexin V-FITC) and propidium iodide (PI) staining. A BD Pharmingen Annexin V-FITC Apoptosis Detection Kit (BD Biosciences, Franklin Lakes, NJ) was used for the apoptosis assay. HeLa cells were placed in a 24-well plate (1 × 106 cells/mL), and after 24 h of incubation, cells were treated with SF-1.5v for 24h and then harvested. After centrifugation, cell pellets were washed twice with cold phosphate-buffered saline (PBS: 137 mM NaCl, 2.7 mM KCl, 10 mM Na_2_HPO_4_, pH 7.4) and suspended in 100 μL of 1 × binding buffer (10 mM HEPES/NaOH, 140 m M NaCl, 2.5 mM CaCl_2_, pH 7.4). Cells were then incubated with 5 μL of annexin V-FITC and 10 μL of PI at room temperature for 15 min in the dark. After incubation, 400 μL of 1 × binding buffer was added to each tube. The cells were immediately analyzed by FACSCalibur flow cytometry (Becton Dickinson, USA).

### 3.5. Western Blotting

HeLa cells at 80% confluence were incubated with SF-1.5v and washed after 24 h in ice-cold PBS and scraped into 200 mL lysis buffer [50 mM Tris-HCl (pH 7.4), 1% Tween 20, 0.25% sodium deoxycholate, 150 mM NaCl, 1 mM EDTA, 1 mM Na_3_VO_4_, 1 mM NaF, and protease inhibitors (1 mg/mL aprotinin, 10 mg/mL leupeptin and 1 mM 4-(2-aminoethyl) benzenesulfonyl fluoride] for 2 h in ice. Protein extracts were cleared by centrifugation and protein concentrations were determined using BCA protein assay kit (Pierce, USA) with bovine serum albumin as standard. An equal volume of sodium dodecyl sulfate (SDS) gel loading buffer [100 mM Tris-HCl (pH 6.8), 200 mM dithiothreitol (DTT), 4% SDS, 0.1% bromophenol blue and 20% glycerol] was added to samples, which were subsequently boiled for 10 min. From each sample, 50 mg of protein was loaded onto SDS-PAGE and blotted onto PVDF membranes (Millipore, Bedford, MA, USA). Membranes were blocked in 1% fat-free dried milk or 2% bovine serum albumin in Tris-buffered saline (TBS) with 0.05% Tween 20 (TBST) and incubated overnight at 4 °C with appropriate primary antibody at 1:1000 dilution. After washing in TBST, membranes were incubated with anti-rabbit horseradish peroxidase-conjugated secondary antibodies, at 1:2000 dilution; in blocking buffer for 1 h. The intensity of the specific immunoreactive bands were detected by enhanced chemiluminescence (ECL), using the manufacturer's protocol (Kirkegared and Perry Laboratories) and quantified by densitometry and expressed as a ratio to actin, as previously described [22].

### 3.6. Statistical Analysis

All data were expressed as mean ± standard deviation. Statistical analysis was done by one-way Anova using the SIGMAStat 2.01 software. Student-Newmans-Keuls post-tests were performed for multiple group comparison. In all cases statistical significance was set at p < 0.05.

## 4. Conclusions

Our studies demonstrate that heterofucan SF-1.5v inhibited growth of the Hela human uterine adenocarcinoma cell line by inducing apoptosis using a mechanism independent of caspases activation. SF-1.5v also induces GSK activation, but this protein is not involved in the heterofucan SF-1.5v-induced apoptosis mechanism. SF-1.5v induces apoptosis mainly by inducing AIF release from mitochondria into cytosol. These data support the hypothesis that SF-1.5v may have potential for treating cervical cancer.
